# CRISPR-Cas and Restriction-Modification Act Additively against Conjugative Antibiotic Resistance Plasmid Transfer in *Enterococcus faecalis*

**DOI:** 10.1128/mSphere.00064-16

**Published:** 2016-06-01

**Authors:** Valerie J. Price, Wenwen Huo, Ardalan Sharifi, Kelli L. Palmer

**Affiliations:** Department of Biological Sciences, The University of Texas at Dallas, Richardson, Texas, USA; University of Nebraska Medical Center

**Keywords:** *Enterococcus*, antibiotic resistance, CRISPR, plasmids, horizontal gene transfer

## Abstract

*Enterococcus faecalis* is an opportunistic pathogen and a leading cause of nosocomial infections. Conjugative pheromone-responsive plasmids are narrow-host-range mobile genetic elements (MGEs) that are rapid disseminators of antibiotic resistance in the *faecalis* species. Clustered regularly interspaced short palindromic repeat (CRISPR)-Cas and restriction-modification confer acquired and innate immunity, respectively, against MGE acquisition in bacteria. Most multidrug-resistant *E. faecalis* isolates lack CRISPR-Cas and possess an orphan locus lacking *cas* genes, CRISPR2, that is of unknown function. Little is known about restriction-modification defense in *E. faecalis*. Here, we explore the hypothesis that multidrug-resistant *E. faecalis* strains are immunocompromised. We assessed MGE acquisition by *E. faecalis* T11, a strain closely related to the multidrug-resistant hospital isolate V583 but which lacks the ~620 kb of horizontally acquired genome content that characterizes V583. T11 possesses the *E. faecalis* CRISPR3-*cas* locus and a predicted restriction-modification system, neither of which occurs in V583. We demonstrate that CRISPR-Cas and restriction-modification together confer a 4-log reduction in acquisition of the pheromone-responsive plasmid pAM714 in biofilm matings. Additionally, we show that the orphan CRISPR2 locus is functional for genome defense against another pheromone-responsive plasmid, pCF10, only in the presence of *cas9* derived from the *E. faecalis* CRISPR1-*cas* locus, which most multidrug-resistant *E. faecalis* isolates lack. Overall, our work demonstrated that the loss of only two loci led to a dramatic reduction in genome defense against a clinically relevant MGE, highlighting the critical importance of the *E. faecalis* accessory genome in modulating horizontal gene transfer. Our results rationalize the development of antimicrobial strategies that capitalize upon the immunocompromised status of multidrug-resistant *E. faecalis*.

**IMPORTANCE** *Enterococcus faecalis* is a bacterium that normally inhabits the gastrointestinal tracts of humans and other animals. Although these bacteria are members of our native gut flora, they can cause life-threatening infections in hospitalized patients. Antibiotic resistance genes appear to be readily shared among high-risk *E. faecalis* strains, and multidrug resistance in these bacteria limits treatment options for infections. Here, we find that CRISPR-Cas and restriction-modification systems, which function as adaptive and innate immune systems in bacteria, significantly impact the spread of antibiotic resistance genes in *E. faecalis* populations. The loss of these systems in high-risk *E. faecalis* suggests that they are immunocompromised, a tradeoff that allows them to readily acquire new genes and adapt to new antibiotics.

## Abstract (precis)

*Enterococcus faecalis* is a bacterium that normally inhabits the gastrointestinal tracts of humans and other animals. Although these bacteria are members of our native gut flora, they can cause life-threatening infections in hospitalized patients. Antibiotic resistance genes appear to be readily shared among high-risk *E. faecalis* strains, and multidrug resistance in these bacteria limits treatment options for infections. Here, we find that CRISPR-Cas and restriction-modification systems, which function as adaptive and innate immune systems in bacteria, significantly impact the spread of antibiotic resistance genes in *E. faecalis* populations. The loss of these systems in high-risk *E. faecalis* suggests that they are immunocompromised, a tradeoff that allows them to readily acquire new genes and adapt to new antibiotics.

## INTRODUCTION

*Enterococcus faecalis* is a Gram-positive bacterium that normally colonizes the gastrointestinal (GI) tracts of humans and other animals (1) and opportunistically colonizes wounds and the bloodstream, leading to the life-threatening infections bacteremia and endocarditis (1–3). Since the 1980s, *E. faecalis* strains have become increasingly associated with nosocomial (hospital-acquired) infections (4–6).

*E. faecalis* appears to have a remarkable propensity for acquisition of antibiotic resistance genes by horizontal gene transfer (HGT). Mobile genetic elements (MGEs) such as conjugative and mobilizable plasmids and transposons are common in *E. faecalis* clinical isolates. They encode resistance to vancomycin, aminoglycosides, tetracycline, chloramphenicol, ampicillin, linezolid, and other antibiotics (7–13). Vancomycin-resistant *E. faecalis* strains are of particular concern and have been deemed serious public health threats by the U.S. Centers for Disease Control and Prevention (14). The emergence of HGT-acquired antibiotic resistance in *E. faecalis* is an ongoing problem that will limit the usefulness of future antibiotics. A unique group of narrow-host-range conjugative plasmids called the pheromone-responsive plasmids (PRPs) are rapid disseminators of antibiotic resistance, cytolytic toxin biosynthesis, and other virulence traits among *E. faecalis* strains but cannot replicate outside the species (8, 15–17). The *in vivo* transfer frequency of PRPs is on the order of one transconjugant per 10 to 100 donor cells (18–20).

Genome analyses indicate that multidrug-resistant (MDR) *E. faecalis* strains are undergoing HGT-driven genome expansion (21–25). Exemplary of this, one-fourth of the 3.36-Mb genome of *E. faecalis* V583, a hospital infection isolate collected in 1987 that was among the first vancomycin-resistant enterococci to be reported (26, 27), was acquired by HGT (23, 26). V583 originates from one of a group of high-risk enterococcal clonal complexes that are associated with nosocomial infections and are commonly resistant to multiple antibiotics (28, 29). In comparison to V583, the genome of the vancomycin-susceptible *E. faecalis* T11 urinary tract isolate, collected in 1992, is only 2.74 Mbp (21, 23). V583 and T11 share 99.5% average nucleotide sequence identity in their core genomes; thus, these strains are very closely related. However, V583 has an additional ~620 kb of HGT-acquired content (21, 30). V583 and T11 are useful comparators for understanding the impacts of HGT on enterococcal biology.

In previous work, we proposed a model for the emergence of MDR, genome-expanded *E. faecalis* strains (30). Our hypothesis is that these strains lack or have lost endogenous barriers to HGT. Antibiotic use inadvertently selects for outgrowth of these immunocompromised strains with enhanced abilities to acquire MGEs, thereby assisting their rapid adaptation to the GI tracts of antibiotic-treated patients and the hospital environment.

Clustered regularly interspaced short palindromic repeat (CRISPR)-Cas systems are genome defense systems that are endogenous barriers to HGT in bacteria. CRISPR loci consist of short repeat sequences interspersed with unique spacer sequences (31, 32). A set of genes encoding nucleases (*cas* genes) are typically located near the CRISPR (33). Type II CRISPR-Cas loci consist of a CRISPR array, the type-specific *cas9* gene, and *cas1* and *cas2* genes (34, 35) (see Fig. S1A in the supplemental material). The mechanism for type II CRISPR-Cas genome defense has been recently reviewed (36) and is summarized here. When cells with type II CRISPR-Cas are challenged with MGEs, some cells incorporate a short segment (protospacer) of the invading MGE genome into the CRISPR as a novel spacer; this is the adaptation phase. By this mechanism, the CRISPR serves as a heritable memory of MGE encounters. Short sequence motifs adjacent to protospacers, called protospacer-adjacent motifs (PAMs), as well as the Cas nucleases are required for adaptation. To provide immunity to MGEs, the CRISPR is transcribed into a pre-CRISPR RNA (pre-crRNA) and processed to mature crRNAs using RNase III, Cas9, and a *trans*-activating crRNA (tracrRNA) that has sequence complementarity to CRISPR repeats. This is the expression phase. If an MGE possessing the protospacer and PAM enters the cell, the Cas9 nuclease is directed to the MGE genome by a crRNA/tracrRNA complex with sequence complementarity to the protospacer. The HNH endonuclease domain of Cas9 cleaves the complementary protospacer strand, and the RuvC endonuclease domain of Cas9 cleaves the noncomplementary protospacer strand, generating a double-stranded DNA (dsDNA) break in the invading MGE. This is the interference phase. In summary, type II CRISPR-Cas systems provide adaptive immunity against MGEs.

10.1128/mSphere.00064-16.1 Figure S1  Representative organization of the type II CRISPR loci found in *E. faecalis*. (A) Type II CRISPR-Cas interference mechanism. The model for type II CRISPR-Cas function shown here has been adapted from a previously proposed model (36). The mechanism is described in three stages: (i) adaptation, (ii) expression, and (iii) interference. During adaptation, a new spacer originating from foreign DNA (red protospacer sequence) is integrated into the leader end of the array. Subsequently, the CRISPR array is transcribed into pre-crRNA; the pre-crRNA and tracrRNA form a complex that is processed by the host RNase III. Another processing event produces the mature crRNA consisting of parts of a spacer and adjacent repeat sequence. Finally, in interference, the mature tracrRNA:crRNA duplex guides Cas9 to the foreign DNA target by base-pairing with a sequence complementary to the spacer and PAM proximity, promoting cleavage of DNA through the two endonuclease domains of Cas9. PAM, protospacer-adjacent motif. (B) Three CRISPR loci identified in *E. faecalis*. Gray arrows represent the location of the CRISPR loci relative to orthologs of the V583 genome. The structures of CRISPR1-*cas* and CRISPR3-*cas* are similar, but the locations of tracrRNA and the sizes of genes within the loci differ; nucleotide length of genes are given within arrows. Designation of *csn2a* or *csn2b* is based on data in reference 65. Download Figure S1, PDF file, 0.05 MB. Copyright © 2016 Price et al. 2016 Price et al. This content is distributed under the terms of the Creative Commons Attribution 4.0 International license.

Two type II CRISPR-Cas systems, called CRISPR1-Cas and CRISPR3-Cas, occur with variable distribution across the *faecalis* species (22, 30, 37–39). There is an additional type II locus, CRISPR2, that lacks associated *cas* genes but whose presence is conserved across the species (see Fig. S1B in the supplemental material) (39). There is a striking relationship between HGT-acquired antibiotic resistance and CRISPR-Cas presence in *E. faecalis*. Specifically, most multidrug-resistant *E. faecalis* strains lack CRISPR-Cas and possess only the orphan CRISPR2 (30, 39). This suggests that CRISPR-Cas systems, by acting as barriers to MGE acquisition, are antagonistic to the evolution of multidrug resistance in *E. faecalis*. However, a role for CRISPR-Cas in *E. faecalis* genome defense has yet to be experimentally demonstrated.

Restriction-modification (R-M) systems provide another form of genome defense by acting as barriers to HGT through self-recognition versus non-self-recognition of methylation signatures. In R-M defense, a cell modifies its “self” DNA at specific sequence motifs. Common modifications conferred by DNA methyltransferases (MTases) are 6-methyladenine (m6A), 4-methylcytosine (m4C), and 5-methylcytosine (m5C) (40). Restriction endonucleases (REases) recognize and degrade nonmodified “non-self” DNA (41, 42). In previous work, we studied R-M systems in the model *E. faecalis* OG1RF strain (43). We determined that *E. faecalis* OG1RF possesses a type II R-M system, EfaRFI, that is capable of providing modest but significant defense against the PRP pCF10 (43). Additional analysis of 17 *E. faecalis* strains revealed that no core R-M systems occur in the species, signifying that these systems occur within the accessory genome of *E. faecalis*.

In this study, we used *E. faecalis* T11 as a model to assess roles of CRISPR3-Cas and the orphan CRISPR2 locus in genome defense against PRPs. We also evaluated synergism between two types of genome defense, R-M and CRISPR-Cas. By using conjugation assays and the model PRPs pAM714 and pCF10, we demonstrated that CRISPR3-Cas is active for sequence-specific genome defense. Our results also demonstrate that, together, CRISPR-Cas and R-M provide additive defense for the cell, with a striking 4-log difference in plasmid acquisition frequencies between strains equipped with or deficient for CRISPR-Cas and R-M defense. Our analysis of the orphan CRISPR2 locus revealed that this locus requires CRISPR1-Cas-encoded factors in order to provide genome defense and cannot provide defense against MGEs on its own. Overall, our results are significant because they support the hypothesis that MDR hospital *E. faecalis* strains are immunocompromised.

## RESULTS

### CRISPR3-Cas is a genome defense system in *E. faecalis*.

**FIG 1:**
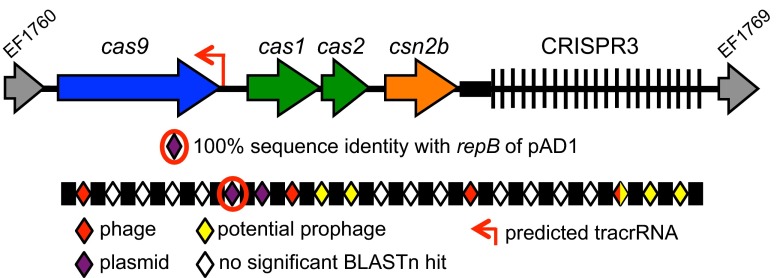
CRISPR3-*cas* locus of *E. faecalis* T11. The CRISPR3 locus of T11 consists of 21 unique spacer sequences of 30 nucleotides (diamonds) flanked by direct repeat sequences of 36 nucleotides each (rectangles); the entire sets of repeats and spacers are expanded below the locus for clarity. Spacers sharing significant identity with MGEs (see Table S2 in the supplemental material) are colored based on the type of genetic element with which they share identity: red, phage; purple, plasmids; yellow, potential prophage. Gray arrows denote V583 gene orthologs. The red arrow between *cas9* and *cas1* represents the predicted location of the CRISPR3 tracrRNA. The black rectangle upstream of the CRISPR array represents the leader region.

*E. faecalis* T11 is closely related to the hospital strain V583 but lacks the multidrug resistance and HGT-driven genome expansion that are characteristic of V583 (21). T11 possesses CRISPR3-Cas and the orphan CRISPR2 (30). Spacer 6 of the T11 CRISPR3 locus is identical to the *repB* sequence from the model 60-kb pheromone-responsive pAD1 plasmid (30). The T11 CRISPR3 locus is shown in Fig. 1, and an analysis of T11 CRISPR3 spacer identities is shown in Table S2 in the supplemental material. By aligning protospacers and adjacent sequences, the CRISPR3 PAM sequence was found to be NNRTA (see Fig. S2 and Table S2).

10.1128/mSphere.00064-16.2 Figure S2  Predicted PAM sequences for the CRISPR loci in *E. faecalis*. Motifs were determined utilizing the MEME motif alignment web server (62) for CRISPR1-Cas (A), CRISPR2 (B), and CRISPR3-Cas (C). Similarity in CRISPR1-Cas and CRISPR2 motifs and a unique CRISPR3-Cas motif are consistent with the differences in the consensus repeat sequences of the three loci. Download Figure S2, PDF file, 0.1 MB. Copyright © 2016 Price et al. 2016 Price et al. This content is distributed under the terms of the Creative Commons Attribution 4.0 International license.

**FIG 2:**
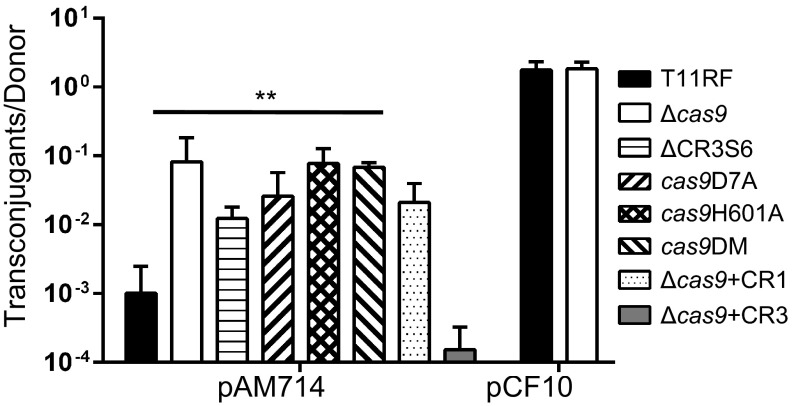
*E. faecalis* CRISPR3-*cas* provides sequence-specific defense against PRPs. Conjugation frequencies between *E. faecalis* OG1SSp harboring either pAM714 (left) or pCF10 (right) and T11RF and its derivatives are indicated. Conjugation frequency data represent ratios of transconjugants to donors in mating reactions. The pAM714 conjugation frequency is significantly higher for recipients that lack *cas9* (Δ*cas9*), lack CRISPR3 spacer 6 (ΔCR3S6), or have mutations in either (*cas9*D7A; *cas9*H601A) or both (*cas9*DM) of the RuvC and HNH endonuclease coding regions of *cas9*. Complementation was observed with CRISPR3 *cas9* (Δ*cas9*+CR3) but not with CRISPR1 *cas9* (Δ*cas9*+CR1)*.* Data represent results of a minimum of 3 independent mating experiments. Significance was assessed using a one-tailed Student’s *t* test; *P* values are relative to T11RF: **, *P* < 0.005.

We tested the hypothesis that T11 CRISPR3-Cas interferes with pAD1 acquisition, using conjugation assays with *E. faecalis* OG1SSp as a plasmid donor and with T11 and its derivatives as plasmid recipients (see Table 1 for a list of plasmids and strains used in this study). T11 was passaged to create a rifampin- and fusidic acid-resistant derivative for use in conjugation experiments (referred to as T11RF). Deletion of CRISPR3 *cas9* from T11RF resulted in a significant increase in acquisition of a pAD1 derivative conferring erythromycin resistance (pAM714 [44, 45]) in plate (biofilm) matings (Fig. 2), providing evidence that CRISPR3-Cas is active for genome defense in this strain. This increase in conjugation frequency was not observed for the 67-kb pheromone-responsive pCF10 plasmid, which is not targeted by CRISPR3 spacers (Fig. 2). We complemented the T11RF CRISPR3 *cas9* deletion with T11 CRISPR3 *cas9* (Δ*cas9*+CR3) at a neutral site on the T11 chromosome. However, complementation was not observed upon integration of *cas9* derived from the *E. faecalis* ATCC 4200 CRISPR1-Cas locus (Δ*cas9*+CR1) (Fig. 2). Deletion of CRISPR3 spacer 6 (ΔCR3S6) resulted in an increase in the conjugation frequency similar to what was observed for the *cas9* deletion, confirming that the CRISPR is required for genome defense. Finally, alignment with the *Streptococcus pyogenes* Cas9 (SpCas9) and *S. aureus* Cas9 (SaCas9) sequences was used to predict the locations of the RuvC and HNH endonuclease domains of *E. faecalis* CRISPR3 Cas9 (EfCR3Cas9; see Fig. S3 in the supplemental material). Single amino acid substitutions were made in these two domains of EfCR3Cas9, generating a D7A substitution in the RuvC-I domain (*cas9*D7A) and an H601A substitution in the HNH domain (*cas9*H601A). These positions correspond to D10 and H557 in SaCas9, for which D10A and H557A substitutions result in a loss of DNA cleavage activity (46), and D10 and H840 in SpCas9, for which D10A substitution results in a loss of protospacer non-complementary-strand cleavage and H840A substitution results in a loss of protospacer complementary-strand cleavage (47). A final strain, *cas9*DM, was generated that possessed both substitutions. Conjugation frequencies obtained with these strains as recipients were similar to those seen with the *cas9* deletion mutant (Fig. 2), implicating these residues as active sites in EfCR3Cas9. Further, that the *E. faecalis* Cas9 D7A and H601A substitutions have equivalent impacts on pAD1 acquisition suggests that pAD1 dsDNA is required for PRP interference by CRISPR3-Cas. These experiments establish that CRISPR3-Cas is a sequence-specific genome defense system in *E. faecalis* T11.

10.1128/mSphere.00064-16.3 Figure S3 Cas9 sequence alignments. *Streptococcus pyogenes* Cas9 (SpCas9) was used as the reference sequence in an alignment with *Staphylococcus aureus* Cas9 (SaCas9) and *E. faecalis* CRISPR3 Cas9 (EfCR3Cas9) proteins. The MUSCLE alignment software was used with default parameters. Active site residues (D10 and H601 for T11) are boxed. Download Figure S3, PDF file, 0.1 MB. Copyright © 2016 Price et al. 2016 Price et al. This content is distributed under the terms of the Creative Commons Attribution 4.0 International license.

**TABLE 1:** Plasmids and strains

Strain or plasmid name	Description	Reference and/or source
*E. coli* strain		
EC1000	Cloning host, providing *repA* in *trans*, for pLT06- and pGEM-T-Easy-derived plasmids	63
*E. coli* plasmids		
pGEM T-Easy	Plasmid containing T-overhangs in MCS,^a^ used for subcloning of DNA fragments for mutant generation in *E. faecalis*	Promega
pLT06	Markerless exchange plasmid; confers chloramphenicol resistance	58
pWH03	Derivative of pLT06 containing OG1RF_11778 and OG1RF_11789 for integration into neutral site on chromosome	43
pVP102	Derivative of pLT06 to create markerless, in-frame deletion of CRISPR3-*cas9* in T11RF	This study
pAS106	Derivative of pLT06 to create deletion of spacer 6 in CRISPR3 locus of T11RF	This study
pVP105	Derivative of pLT06 to change amino acid 7 of T11 CRISPR3 Cas9 from aspartic acid to alanine	This study
pG19	Derivative of pWH03 to integrate the CRISPR1-*cas9* gene, its native promoter, and predicted tracrRNA into the T11 chromosome between EFMG_00904 and EFMG_00905	This study
pVP301	Derivative of pWH03 to integrate the CRISPR3-*cas9* gene, its native promoter, and predicted tracrRNA into the T11 chromosome between EFMG_00904 and EFMG_00905	This study
pWH01	Derivative of pLT06 to create markerless, in-frame deletion of OG1RF_11621-OG1RF_11622 in OG1SSp	43
pWH43	Derivative of pWH03 to integrate OG1SSp OG1RF_11621-OG1RF_11622 and its native promoter into the chromosome between OG1RF_11778 and OG1RF_11789	This study
pVP401	Derivative of pGEM-T-Easy with 100-bp insert, including T11 CRISPR2 spacer 1 and the consensus CRISPR2 PAM	This study
pVP107	Derivative of pLT06 to knock-in the T11 CRISPR2 spacer 1 sequence and consensus CRISPR2 PAM into the *uvrB* gene of pCF10	This study
pVP402	Derivative of pGEM-T-Easy with 100-bp insert, including T11 CRISPR2 spacer 1 and the consensus CRISPR3 PAM	This study
pVP108	Derivative of pLT06 to knock-in T11 CRISPR2 spacer 1 and the consensus CRISPR3 PAM into the *uvrB* gene of pCF10	This study
pVP109	Derivative of pLT06 to change amino acid 601 of T11 CRISPR3 Cas9 from histidine to alanine	This study
*E. faecalis* strains		
T11RF	Rifampin-fusidic acid-resistant derivative of T11	23 and this study
T11RFΔ*cas9*	T11RF CRISPR3*-cas9* deletion mutant	This study
T11RFΔ*cas9*+CR3	T11RFΔ*cas9* mutant with chromosomal integration of CRISPR3 *cas9* between EFMG_00904 and EFMG_00905	This study
T11RFΔ*cas9*+CR1	T11RFΔ*cas9* mutant with chromosomal integration of CRISPR1 *cas9* and the predicted CRISPR1 tracrRNA between EFMG_00904 and EFMG_00905	This study
T11RFΔCR3S6	T11RF with a deletion of CRISPR3 spacer 6	This study
T11RF*cas9*D7A	T11RF with chromosomal mutation in the RuvC nuclease coding region of *cas9*	This study
T11RF*cas9*H601A	T11RF with chromosomal mutation in the HNH nuclease coding region of *cas9*	This study
T11RF*cas9*DM	T11RF with chromosomal mutations in the predicted RuvC and HNH nuclease coding regions of *cas9*	This study
OG1SSp pAM714	Spectinomycin-streptomycin-resistant derivative of OG1 harboring pAM714, an erythromycin (carried on Tn*917*)-resistant derivative of pAD1	44, 45
VP701	OG1SSp pAM714 EfaRFI deletion mutant	This study
WH702	VP701 with chromosomal integration of EfaRFI (OG1RF_11621-OG1RF_11622) and its native promoter between OG1RF_11778 and OG1RF_11789	This study
OG1SSp pCF10	Spectinomycin-streptomycin-resistant derivative of OG1 harboring pCF10 encoding tetracycline resistance on Tn*925*	64
VP703	OG1SSp pCF10 EfaRFI deletion mutant	This study
OG1SSp pVP501	OG1SSp pCF10 with insertion of T11 CRISPR2 spacer 1 and consensus CRISPR2 PAM into *uvrB* of pCF10	This study
OG1SSp pVP502	OG1SSp pCF10 with insertion of T11 CRISPR2 spacer1 and consensus CRISPR3 PAM into *uvrB* of pCF10	This study

a MCS, multiple-cloning site.

### Relative contributions of R-M and CRISPR-Cas in defense in *E. faecalis* T11.

**FIG 3:**
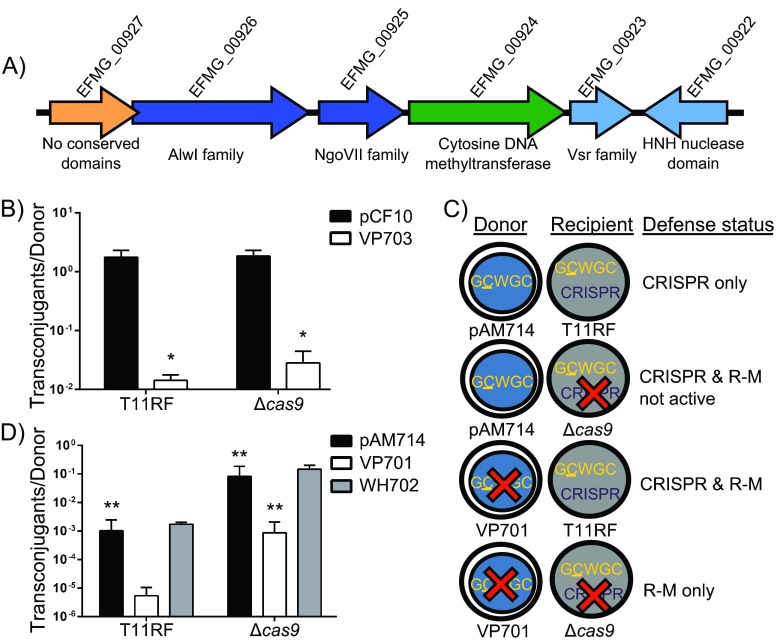
CRISPR-Cas and R-M provide additive defense against PRPs in *E. faecalis*. (A) Organization of the predicted R-M locus of T11; multiple predicted REases are encoded near the MTase. (B) Conjugation frequencies with T11RF and T11RFΔ*cas9* strains as recipients in mating reactions with OG1SSp pCF10 and VP703 as donors. *P* values are relative to transfer of OG1SSp pCF10 to T11RF: *, *P* < 0.05. (C) Schematic representing donor and recipient strains used to assess the individual and collective contributions of R-M and CRISPR-Cas to genome defense. (D) Conjugation frequencies with T11RF and T11RFΔ*cas9* strains as recipients (*x* axis) and with OG1SSp pAM714 (black columns), OG1SSp pAM714 ΔEfaRFI (VP701; white columns), and OG1SSp pAM714 ΔEfaRFI + EfaRFI (WH702; gray columns) as donors. Frequencies are shown as the ratios of transconjugants to donors. Results of these experiments show that the combined effects of CRISPR-Cas and R-M outweigh the effect of either system alone. Data represent results of a minimum of three independent conjugations for all experiments shown. *P* values are relative to transfer of pAM714 from VP701 to T11RF: **, *P* < 0.005. Significance in the data in panels B and D was assessed using a one-tailed Student’s *t* test.

In a previous study, we determined that the genomes of *E. faecalis* OG1RF, OG1SSp, and T11 are modified by 5′-G^m5^CWGC-3′ (43). Deletion of EfaRFI, the R-M system responsible for 5′-G^m5^CWGC-3′ modification in OG1RF and OG1SSp, significantly but modestly (~3-fold) reduced the frequency of pCF10 conjugation between OG1RF mutant cells and OG1SSp (43). Using the strategy for MTase identification that we used in our previous study, we predicted only one MTase in the T11 genome (EFMG_00924), and it has 56% amino acid sequence identity with the EfaRFI MTase (M.EfaRFI) (see Table S3 in the supplemental material). We infer that this MTase is responsible for the 5′-G^m5^CWGC-3′ DNA modification observed for T11 (43). However, the prediction of the corresponding REase for the T11 M.EfaRFI homolog is not straightforward, as there are four genes surrounding the MTase that have conserved endonuclease domains, three of which are predicted to recognize ^m5^C signatures (Fig. 3A; see also Table S3) and only one of which has high amino acid sequence identity with R.EfaRFI (EFMG_00925; 43% identity). Analysis of the *faecalis* pan-genome revealed that this region occurs in a subset of strains with available genome sequence (*E. faecalis* T11, B301, B345, B347, and T19). Synteny analyses performed with T11 and V583 suggest that these accessory genes were displaced in V583 by a transposon carrying the *vanB* vancomycin resistance cassette (23, 26).

The impact of DNA modification on plasmid transfer into T11 was assessed by conjugative transfer of pCF10 from OG1SSp donor strains with (OG1SSp pCF10) or without (VP703) EfaRFI. For OG1SSp pCF10 donors, the plasmid is modified by 5′-G^m5^CWGC-3′ and should be recognized as “self” by the T11 R-M system. For VP703 donors, the plasmid is not modified by 5′-G^m5^CWGC-3′ and should be recognized as “non-self” by the T11 R-M system. Abolishment of DNA modification in the donor strain resulted in a 124-fold reduction in pCF10 plasmid transfer into T11 (Fig. 3B). This effect is much more pronounced than the 3-fold decrease in pCF10 transfer observed in a previous study for the EfaRFI system (43), suggesting that the T11 R-M system possesses features that provide more robust genome defense than EfaRFI.

Next, we sought to determine whether CRISPR-Cas and R-M confer additive genome defense effects in *E. faecalis* T11. pAM714 possesses 59 GCWGC motifs, none of which overlap the protospacer and PAM sequences in *repB*. pAM714 is expected to be modified with 5′-G^m5^CWGC-3′ by OG1SSp donor strains. For the experiments whose results are shown in Fig. 2, pAM714 transferred from OG1SSp to T11 was modified by 5′-G^m5^CWGC-3′ and recognized as “self” DNA by the T11 R-M system. Therefore, CRISPR3-Cas but not R-M defense was active under that condition. We modulated self-signals versus non-self-signals at 5′-GCWGC-3′ motifs in the donor strain to determine the individual and collective impacts of R-M and CRISPR-Cas defense on pAM714 acquisition. The design of these experiments is shown in Fig. 3C. The donor strains used were OG1SSp pAM714, an OG1SSp pAM714 derivative with a deletion of EfaRFI (strain VP701), and a VP701 complement strain with EfaRFI genes integrated into a neutral site on the chromosome (WH702). When both CRISPR-Cas defense and R-M defense are active, the average conjugation frequency (expressed as transconjugants/donors) is 5.4 × 10^−6^; we used this value as a reference for comparisons (Fig. 3D). When CRISPR-Cas defense has been compromised by the loss of *cas9* but R-M defense is active, the average conjugation frequency is 8.7 × 10^−4^, a 160-fold increase in plasmid transfer. When R-M defense is not active due to the incoming plasmid being modified as “self” but CRISPR-Cas defense is active, the average conjugation frequency is 1 × 10^−3^, a 188-fold increase in plasmid transfer. When neither defense system is active, the average conjugation frequency is 8.25 × 10^−2^, a 15,277-fold increase in plasmid transfer. Overall, we conclude that R-M and CRISPR-Cas, both individually and collectively, have significant impacts on conjugative plasmid transfer in *E. faecalis* T11.

### T11 CRISPR2 does not provide genome defense unless CRISPR1 Cas9 is present.

An orphan CRISPR locus lacking *cas* genes and with various configurations of spacers, called CRISPR2, occurs in all *E. faecalis* genomes, including multidrug-resistant strains (30, 39). The consensus repeats of CRISPR2 and CRISPR1-Cas are identical, suggesting that they are functionally linked (see Fig. S4 in the supplemental material). The repeat sequences of CRISPR3 are only 58% identical to those of CRISPR1/CRISPR2 (see Fig. S4). In previous work, we hypothesized that CRISPR2 is inactive for genome defense in strains lacking CRISPR1-Cas, i.e., high-risk lineages (30). An alternative hypothesis that would explain the conservation of CRISPR2 is that CRISPR2 confers genome defense by a Cas-independent mechanism. We used T11 as a model strain to determine whether CRISPR2 can confer genome defense alone or in conjunction with CRISPR-Cas-encoded factors.

10.1128/mSphere.00064-16.4 Figure S4 Alignment of direct repeat sequences of the CRISPR loci found in *E. faecalis*. Consensus repeat sequences from each of the CRISPRs were aligned using Geneious. CRISPR1-Cas and CRISPR2 repeats are identical, whereas the CRISPR3 repeat shares only 58% identity with them. Repeats were derived from the following genomes: for CRISPR1-Cas, OG1RF; CRISPR2, and OG1RF; for CRISPR3-Cas, T11. Download Figure S4, PDF file, 0.1 MB. Copyright © 2016 Price et al. 2016 Price et al. This content is distributed under the terms of the Creative Commons Attribution 4.0 International license.

**FIG 4:**
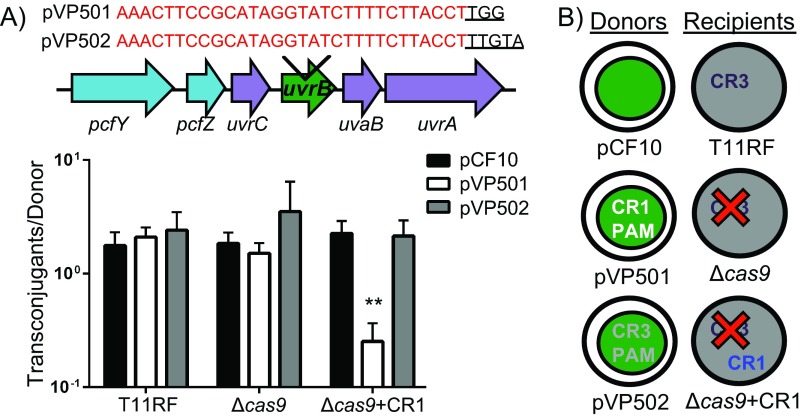
Orphan CRISPR2 provides defense against PRPs in the presence of CRISPR1 *cas9*. (A) Top panel: schematic of how the T11 CRISPR2 spacer 1 sequence and corresponding PAM sequences (underlined) were introduced into pCF10. (Bottom panel) Conjugation frequencies of T11RF and its derivatives as recipient strains in conjugation with OG1SSp harboring pCF10, pVP501, and pVP502. The T11 CRISPR2 locus provides genome defense against pVP501 in the presence of CRISPR1 *cas9* and its predicted tracrRNA. Results also demonstrate CRISPR1 *cas9* PAM specificity to the NGG sequence. A minimum of three independent conjugation reactions are represented. Significance was assessed using a one-tailed Student’s *t* test; *P* values are relative to pVP501 transfer to Δ*cas9*+CR1: **, *P* < 0.005. (B) Outline of donor and recipient strains used for assessing the function of CRISPR2.

The spacer content of CRISPR1 and CRISPR2 loci of six *E. faecalis* strains was used to determine their respective PAM sequences, which are predicted to be identical (NGG; see Fig. S2 and Table S2 in the supplemental material). The CRISPR2 of T11 possesses 4 spacers that lack identity to known MGEs but that are identical to spacers that occur in CRISPR2 loci of other *E. faecalis* strains, two of which are present in the CRISPR2 of V583 (39). We inserted a protospacer identical to T11 CRISPR2 spacer 1, along with an NGG PAM sequence (for CR1 and CR2) or an NNRTA PAM sequence (for CRISPR3; see Fig. S2 and Table S2), into pCF10, generating pVP501 or pVP502, respectively (Fig. 4A; see also Fig. S5). The integration of the same protospacer with either of two different PAM sequences was performed to assess Cas9 specificity with respect to its cognate target recognition motif. We then evaluated conjugative transfer of these two plasmids and wild-type pCF10 from OG1SSp to T11RF and its derivatives (Fig. 4B).

10.1128/mSphere.00064-16.5 Figure S5  Construction of pCF10 derivatives pVP501 and pVP502. Download Figure S5, PDF file, 0.1 MB. Copyright © 2016 Price et al. 2016 Price et al. This content is distributed under the terms of the Creative Commons Attribution 4.0 International license.

As previously shown (Fig. 2 and 3), there was no significant change in the conjugation frequency of pCF10 between T11RF and the T11RFΔ*cas9* mutant (Fig. 4A, bottom panel). Moreover, the addition of the T11 CRISPR2 spacer 1 and PAM sequences into pCF10 had no effect on conjugation frequency in T11RF or the T11RFΔ*cas9* mutant. We conclude that under these conditions, CRISPR2 alone cannot provide defense in the presence of a protospacer target and the predicted PAM. We then set out to determine if the presence of the *E. faecalis* CRISPR1 *cas9* and its predicted tracrRNA would impact plasmid transfer. To test this, we integrated the CRISPR1 *cas9* gene and the predicted tracrRNA coding regions into a neutral site on the T11RFΔ*cas9* mutant chromosome. A 6-fold reduction in conjugation frequency was observed between mating of pVP501 to the T11RF Δ*cas9* mutant and mating of pVP501 to the T11RFΔ*cas9*+CR1 mutant, revealing that CRISPR2 requires CRISPR1-Cas factors to provide genome defense. Finally, no change in conjugation frequency was observed when using pVP502. This result, in conjunction with observing a similar conjugation frequency of pVP502 into T11RF, provides experimental evidence that supports the prediction of the PAM for CRISPR1/CRISPR2. These results demonstrate a functional linkage between CRISPR1-Cas and CRISPR2 through CRISPR1-Cas-encoded factors.

## DISCUSSION

A correlation between the lack of CRISPR-Cas and multidrug resistance in *E. faecalis* has been previously established using genome analysis (30). The aim of the current work was to experimentally assess genome defense strategies in *E. faecalis* using clinically relevant conjugative plasmids as model MGEs. Broadly, the results of our study illustrate the importance of the variable genome of *E. faecalis*. We explored genome defense in *E. faecalis* T11, a strain closely related to the high-risk MDR strain V583. Two components of the *faecalis* variable genome that occur in T11 but are absent from V583, CRISPR3-Cas and a predicted R-M system, have a combined 4-log impact on the conjugative transfer of the pheromone-responsive pAM714 plasmid in biofilm settings. These results substantiate our hypothesis that high-risk *E. faecalis* strains have readily acquired resistance to antibiotics due to their lack of genome defense. In future work, it will be of interest to assess the kinetics of CRISPR-Cas and R-M defense against antibiotic resistance plasmids, as well as their comparative efficiencies in providing genome defense in biofilm, planktonic, and polymicrobial settings.

Our work demonstrated that the orphan CRISPR2 locus in T11 does not confer genome defense in the absence of CRISPR1-Cas-encoded factors. This is significant because all high-risk, MDR *E. faecalis* strains possess orphan CRISPR2 loci. The conservation of CRISPR2 among *E. faecalis* strains lacking CRISPR1-Cas remains to be explained. CRISPR2 may be maintained in the species by providing another function for the cell, perhaps by acting as a noncoding regulatory RNA. Indeed, both CRISPR2 and a transcript antisense to CRISPR2 have been detected in transcriptome studies of V583 (48, 49), demonstrating that this region is transcriptionally active in the absence of CRISPR1-Cas. There is a precedent for a role for orphan CRISPR loci in regulation of gene expression; the orphan CRISPR *rliB* in *Listeria monocytogenes* regulates expression of *feoAB* (ferrous iron acquisition genes) and impacts virulence (50, 51). This locus undergoes an alternative processing pathway involving polynucleotide phosphorylase (PNPase) (52); therefore, a requirement for host-encoded factors beyond RNase III in *E. faecalis* CRISPR2 function cannot be ruled out. Studies of the V583 CRISPR2 locus are of interest for future work. Of particular interest is testing whether the reintroduction of CRISPR1-Cas into high-risk MDR *E. faecalis* leads to CRISPR adaptation against endogenous MGEs and genome reduction when antibiotic selection is absent.

Although CRISPR3-Cas had a significant impact on conjugation frequency, it was not a perfect barrier to plasmid transfer, as some transconjugants were obtained in every mating reaction. This suggests that a subset of recipient cells have mutations in CRISPR3-Cas that inactivate defense, or that pAM714 plasmids have mutations in the *repB* protospacer or PAM, or perhaps that pAD1 has a mechanism for actively evading CRISPR-Cas defense in a subset of cells. Whether CRISPR-Cas is equally expressed in all recipient cells and how the system is regulated are also unknown. Interestingly, high frequencies of CRISPR-Cas mutations have been observed in other type II CRISPR systems (53, 54). Further analysis of these “escaper” transconjugants will be the focus of future work. Importantly, R-M defense can still impede plasmid transfer in CRISPR-Cas mutant cells. Our observation that CRISPR-Cas defense and R-M defense individually contribute significantly to anti-plasmid genome defense is consistent with a previous report that the two modes of defense work additively against phage infection in *Streptococcus thermophilus* (55).

How can this information be applied? Our work supports the development of antimicrobial strategies that monopolize the immunocompromised status of high-risk, MDR *E. faecalis*. These applications include phage therapy and preprogrammed CRISPR-Cas9 systems, introduced by phagemids, that target the bacterial chromosome for destruction (56, 57). These strategies could be used for surface and gastrointestinal tract decolonization of problematic *E. faecalis*. Critical to the success of these strategies will be a greater understanding of *E. faecalis* phage biology, about which little is known, as well as of the potential for Cas9-directed chromosome cleavage in *E. faecalis*.

## MATERIALS AND METHODS

### Bacteria and reagents used.

Strains and plasmids used in this study are shown in Table 1. *E. faecalis* T11RF, a rifampin- and fusidic acid-resistant derivative of *E. faecalis* T11, was isolated by sequential exposure to the antibiotics at 50 µg/ml and 25 µg/ml, respectively. *E. faecalis* strains were cultured in brain heart infusion (BHI) broth or agar at 37°C, unless otherwise stated. Antibiotic concentrations for *E. faecalis* were as follows: rifampin, 50 µg/ml; fusidic acid, 25 µg/ml; spectinomycin, 500 µg/ml; streptomycin, 500 µg/ml; chloramphenicol, 15 µg/ml; tetracycline, 10 µg/ml; erythromycin, 50 µg/ml. *Escherichia coli* strains were cultured in lysogeny broth (LB) with aeration at 225 rpm or LB agar at 37°C, unless otherwise stated. The antibiotic concentration for *E. coli* was as follows: chloramphenicol, 15 µg/ml. Antibiotics were purchased from Sigma-Aldrich. Restriction enzymes were purchased from New England Biolabs and used according to manufacturer protocols. Routine PCR analysis was performed using *Taq* polymerase (New England Biolabs). PCR for cloning procedures utilized Phusion polymerase (Fisher Scientific). Plasmid isolation was performed using a GeneJET Plasmid Miniprep kit (Thermo Scientific). PCR products and restriction digestion reaction mixtures were purified using a GeneJET PCR Purification kit (Thermo Scientific). DNA sequencing was performed at the Massachusetts DNA Core Facility (Boston, MA). Primers used in this study are shown in Table S1 in the supplemental material.

10.1128/mSphere.00064-16.6 Table S1  Primers used in this study. Italicized bases represent restriction enzyme sites used for cloning. Underlined bases show the amino acid change for generation of T11RF*cas9*D7A and T11RF*cas9*H601A as well as the CRISPR1/CRISPR2 PAM or CRISPR3 PAM sequence used for studying CRISPR2 in genome defense. Download Table S1, XLSX file, 0.04 MB. Copyright © 2016 Price et al. 2016 Price et al. This content is distributed under the terms of the Creative Commons Attribution 4.0 International license.

10.1128/mSphere.00064-16.7 Table S2  MGE identities of spacers used to determine PAMs for the three CRISPR loci of *E. faecalis*. The superscript “a” indicates spacer numbers that correspond to a previously published *E. faecalis* CRISPR2 spacer dictionary (39). The superscript “b” indicates protospacer-adjacent sequences extracted from representative hits used to create a putative PAM for each CRISPR locus (Fig. S2). The superscript “c” indicates protospacers that align within 18 kb of each other on the D32 genome; this region could be a prophage or pathogenicity island. Download Table S2, XLSX file, 0.05 MB. Copyright © 2016 Price et al. 2016 Price et al. This content is distributed under the terms of the Creative Commons Attribution 4.0 International license.

10.1128/mSphere.00064-16.8 Table S3  Protein features around the predicted methyltransferase in the T11 R-M region. In addition to strain T11, this configuration of genes occurs in the following *E. faecalis* strains: B301, B345, B347, and T19. Download Table S3, XLSX file, 0.05 MB. Copyright © 2016 Price et al. 2016 Price et al. This content is distributed under the terms of the Creative Commons Attribution 4.0 International license.

### Spacer analysis of *E. faecalis* T11.

The T11 CRISPR3-*cas* and CRISPR2 spacer sequences were used as queries in BLASTn analysis against the NCBI nonredundant nucleotide database. A significance threshold of 86% sequence identity, which allows four mismatches between the query and subject, was used to identify protospacer candidates.

### Generation of T11RF strains used in this study.

In-frame deletions of CRISPR3 *cas9* and CRISPR3 spacer 6 were generated using a previously established protocol (58). Briefly, ~1-kb regions up- and downstream of *cas9* or CRISPR3 spacer 6 in *E. faecalis* T11RF were amplified, digested, and ligated into pLT06 (58) to generate pVP102 and pAS106, respectively. The resulting plasmids were transformed into competent T11RF cells via electroporation (59) and cultured at the permissive temperature of 30°C. Following transformation, a shift to the nonpermissive temperature of 42°C and counterselection on p-chloro-phenylalanine were performed to generate in-frame, markerless deletions. The predicted RuvC and HNH nuclease domain coding regions of CRISPR3 *cas9* were mutated such that residues D7 and H601 were changed to alanine. This was accomplished by amplifying ~1-kb arms up- and downstream of the codons for the 7th and 601st amino acids, but instead of using a restriction site to connect the two arms, overlapping sequences on the internal primers were used to generate the amino acid coding change (underlined in Table S1 in the supplemental material), generating T11RF*cas9*D7A and T11RF*cas9*H601A. Sequencing was used to confirm all modified regions.

Complementation of the *cas9* deletion was accomplished by integrating the gene into a neutral site on the T11 chromosome at a location between open reading frames (ORFs) EFMG_00904 and EFMG_00905. pWH03, a derivative of pLT06 containing ~1-kb arms corresponding to the genes at this site, was used as the backbone vector for insertion of T11 CRISPR3 *cas9* (pVP301) as well as ATCC 4200 CRISPR1 *cas9* (pG19) into the T11RFΔ*cas9* strain. The putative promoter and predicted tracrRNA were included in the complementation constructs for both CRISPR3 *cas9* and CRISPR1 *cas9*, generating strains T11RFΔ*cas9*+CR3 and T11RFΔ*cas9*+CR1; the entire integrated region was confirmed by sequencing.

### Generation of OG1SSp mutants.

The EfaRFI R-M system was deleted in OG1SSp pAM714 using the pLT06 derivative pWH01, as in previous work (43); the deletion was confirmed by sequencing, resulting in strain VP701. Complementation was performed via knock-in of EfaRFI at the neutral locus. Briefly, OG1RF_11622-OG1RF_11621, including its putative promoter region, was ligated into pWH03, resulting in pWH43. pWH43 was electroporated into competent VP701 cells, and temperature shift and counterselection were used as described above to generate WH702; the insertion was confirmed by sequencing.

### Generation of pCF10 mutants.

To insert the T11 CRISPR2 spacer 1 sequence into pCF10, 100-bp single-stranded DNA oligonucleotides were annealed to each other to generate dsDNA. The 100-bp oligonucleotides included sequence from pCF10 *uvrB*, the spacer 1 sequence, and either a CRISPR1/2 PAM or CRISPR3 PAM. Annealed oligonucleotides were subcloned into pGEM T-Easy vector (Promega) for amplification and ligation into pLT06 derivatives designed to insert these sequences into the *uvrB* gene of pCF10 by homologous recombination. See Fig. S5 in the supplemental material for a schematic of constructs used to generate strains OG1SSp pVP501 and OG1SSp pVP502.

### R-M system prediction in T11.

*E. faecalis* T11 contigs were downloaded from the Broad Institute (Enterococcus I Initiative; www.broadinstitute.org) and annotated using RAST (60, 61). Protein sequences were blasted against the NEB rebase gold standards list. Using a bit score cutoff of 60 for MTase identity to the gold standard list, we predicted only one MTase in T11 (EFMG_00924), which is also a homolog of M.EfaRFI (sequence identity, 56%; query coverage, 93%; E value, 2E^−125^).

### Conjugation experiments.

For all conjugation reactions, donor and recipient strains were cultured overnight in BHI broth without antibiotic selection. The next day, cultures were diluted 1:10 into fresh BHI broth and incubated at 37°C for 1.5 h. Next, a 100-µl volume of donor culture was mixed with a 900-µl volume of recipient culture and the mixture was pelleted at 13,000 rpm for 1 min. A 100-µl volume of supernatant was used to resuspend the pellet, which was then plated on BHI agar and incubated at 37°C for 18 h. Cells were collected from the plate with 2 ml 1× PBS supplemented with 2 mM EDTA. Dilutions were plated on BHI agar plates supplemented with antibiotics to quantify donor (spectinomycin and streptomycin with either erythromycin or tetracycline), recipient (rifampin and fusidic acid), or transconjugant (rifampin and fusidic acid with either erythromycin or tetracycline) populations. Plates were incubated for 36 to 48 h at 37°C to allow colonies to develop. Plates with 30 to 300 colonies were used to calculate CFU counts per milliliter. Conjugation frequency was determined by dividing the number of transconjugants by the number of donors.

### PAM identification.

Strains with complete CRISPR arrays (no sequence gaps) were used to identify putative PAMs for the three *E. faecalis* CRISPR loci*.* Protospacers were identified as described above. A total of 15 nucleotides downstream of the protospacer sequence were extracted and subjected to motif detection using MEME (62). The same CRISPR2 spacer sequences often occur in multiple strains (39); therefore, spacer hits to CRISPR2 loci were manually curated from the analysis so that a CRISPR2 spacer was not overrepresented.
