# Supplementary figures and images for: CRISPR-Cas and Restriction-Modification Act Additively against Conjugative Antibiotic Resistance Plasmid Transfer in Enterococcus faecalis
Source: mSphere. 2016 Jun 1;1(3):e00064-16. doi: 10.1128/mSphere.00064-16 (PMC4894674; doi:10.1128/mSphere.00064-16)

A)

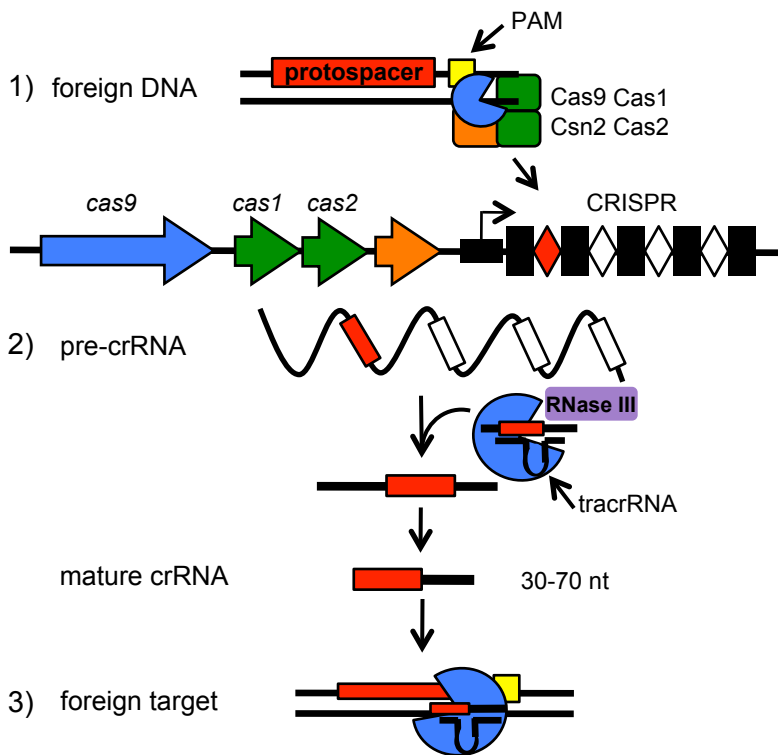

B)

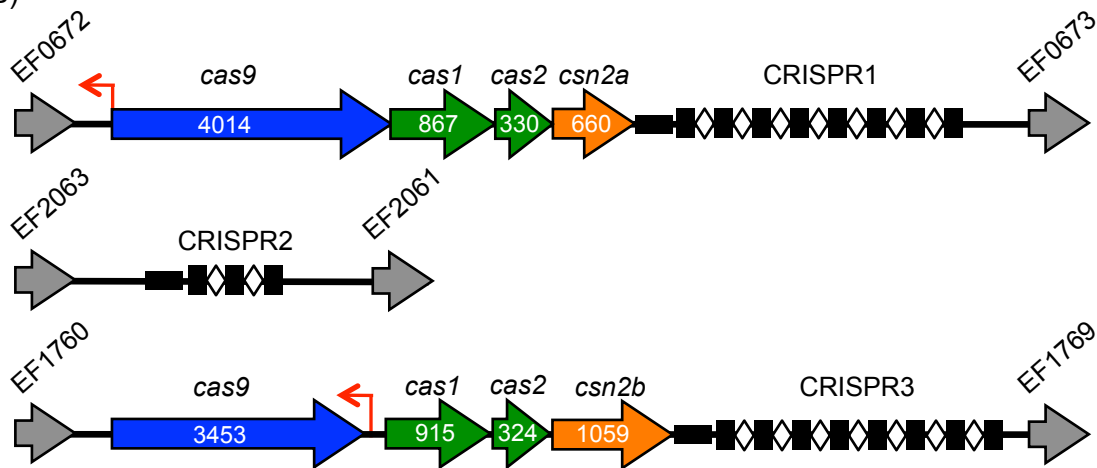

Supplement: Figure S1 [file sph003162100sf4.pdf]

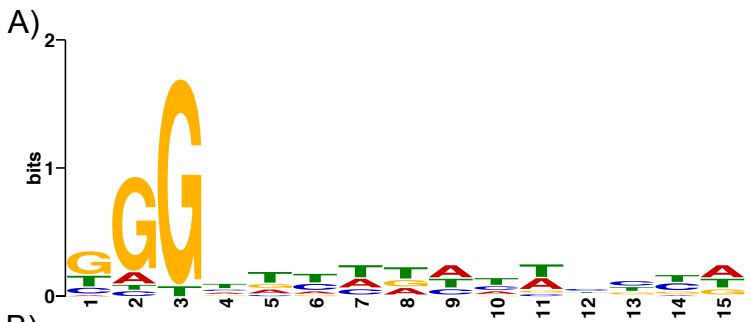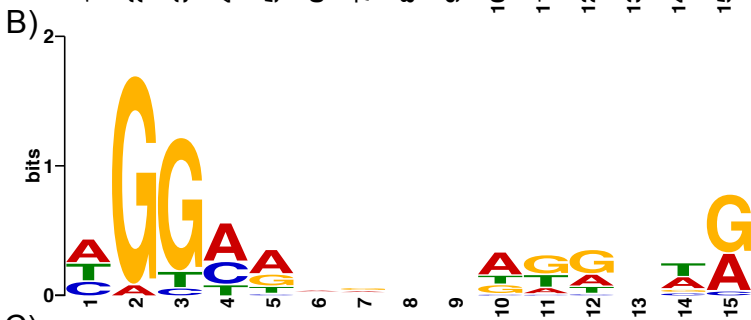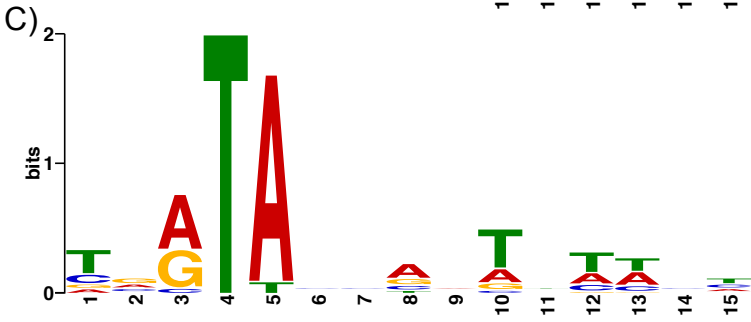

Supplement: Figure S2 [file sph003162100sf5.pdf]
