# Supplementary material for: CRISPR-Cas and Restriction-Modification Act Additively against Conjugative Antibiotic Resistance Plasmid Transfer in Enterococcus faecalis
Source: mSphere. 2016 Jun 1;1(3):e00064-16. doi: 10.1128/mSphere.00064-16 (PMC4894674; doi:10.1128/mSphere.00064-16)
Supplement: Figure S4 [file sph003162100sf7.pdf]

	1 ... 10 ... 20 ... 30 ... 36
**CRISPR1**	GTTTTAGAGTCATGTTGTTTAGAATGGTACCAAAAC
**CRISPR2**	GTTTTAGAGTCATGTTGTTTAGAATGGTACCAAAAC
**CRISPR3**	GTTTTACTGATAAGAAATTATTGAGAGTACAAAAAC
