# Supplementary material for: CRISPR-Cas and Restriction-Modification Act Additively against Conjugative Antibiotic Resistance Plasmid Transfer in Enterococcus faecalis
Source: mSphere. 2016 Jun 1;1(3):e00064-16. doi: 10.1128/mSphere.00064-16 (PMC4894674; doi:10.1128/mSphere.00064-16)
Supplement: Figure S5 [file sph003162100sf8.pdf]

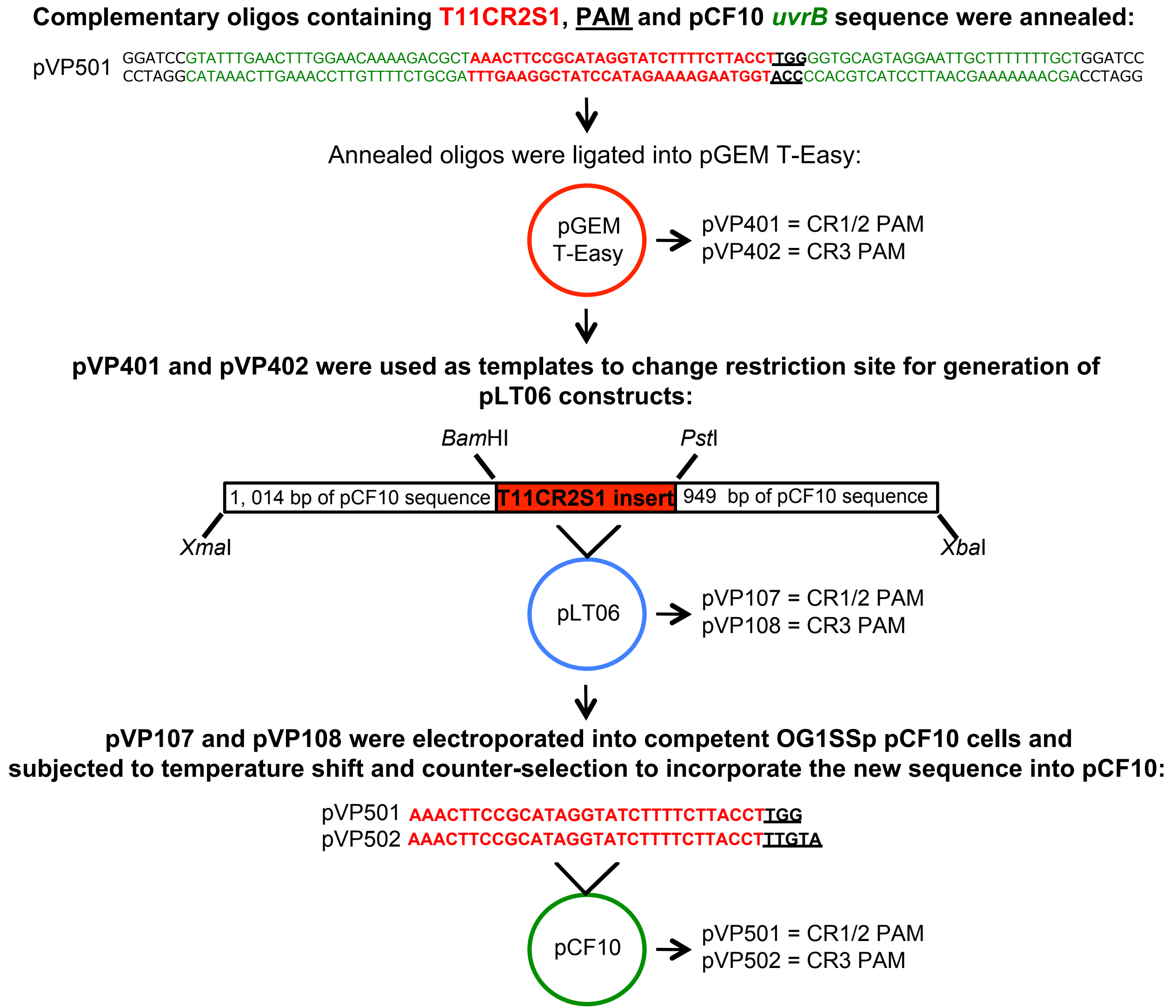

Complementary oligos containing T11CR2S1, PAM and pCF10 uvrB sequence were annealed:
pVP501
GGATCCGTATTTGAACTTTGGAACAAAAGACGCTAAACTTCCGCATAGGTATCTTTTCTTACCTTGGGGTGCAGTAGGAATTGCTTTTTTTGCTGGATCC
CCTAGGCATAAACTTGAAACCTTGTTTTCTGCGATTTGAAGGCTATCCATAGAAAAGAATGGTACCCCACGTCATCCTTAACGAAAAAAACGACCTAGG
Annealed oligos were ligated into pGEM T-Easy:
pGEM T-Easy
pVP401 = CR1/2 PAM
pVP402 = CR3 PAM
pVP401 and pVP402 were used as templates to change restriction site for generation of pLT06 constructs:
BamHI
PstI
1, 014 bp of pCF10 sequence
T11CR2S1 insert
949 bp of pCF10 sequence
XmaI
XbaI
pLT06
pVP107 = CR1/2 PAM
pVP108 = CR3 PAM
pVP107 and pVP108 were electroporated into competent OG1SSp pCF10 cells and subjected to temperature shift and counter-selection to incorporate the new sequence into pCF10:
pVP501 AAACTTCCGCATAGGTATCTTTTCTTACCTTGG
pVP502 AAACTTCCGCATAGGTATCTTTTCTTACCTTTGTA
pCF10
pVP501 = CR1/2 PAM
pVP502 = CR3 PAM
